# Plasma mitochondrial DNA is elevated in obese type 2 diabetes mellitus patients and correlates positively with insulin resistance

**DOI:** 10.1371/journal.pone.0222278

**Published:** 2019-10-10

**Authors:** Larysa V. Yuzefovych, Viktor M. Pastukh, Mykhaylo V. Ruchko, Jon D. Simmons, William O. Richards, Lyudmila I. Rachek

**Affiliations:** 1 Department of Pharmacology, College of Medicine, University of South Alabama, Mobile, Alabama, United States of America; 2 Department of Surgery, College of Medicine, University of South Alabama, Mobile, Alabama, United States of America; East Tennessee State University, UNITED STATES

## Abstract

Cells damaged by mechanical or infectious injury release proinflammatory mitochondrial DNA (mtDNA) fragments into the circulation. We evaluated the relation between plasma levels of mtDNA fragments in obese type 2 diabetes mellitus (T2DM) patients and measures of chronic inflammation and insulin resistance. In 10 obese T2DM patients and 12 healthy control (HC) subjects, we measured levels of plasma cell-free mtDNA with quantitative real-time polymerase chain reaction, and mtDNA damage in skeletal muscle with quantitative alkaline Southern blot. Also, markers of systemic inflammation and oxidative stress in skeletal muscle were measured. Plasma levels of mtDNA fragments, mtDNA damage in skeletal muscle and plasma tumor necrosis factor α levels were greater in obese T2DM patients than HC subjects. Also, the abundance of plasma mtDNA fragments in obese T2DM patients levels positively correlated with insulin resistance. To the best of our knowledge, this is the first published evidence that elevated level of plasma mtDNA fragments is associated with mtDNA damage and oxidative stress in skeletal muscle and correlates with insulin resistance in obese T2DM patients. Plasma mtDNA may be a useful biomarker for predicting and monitoring insulin resistance in obese patients.

## Introduction

Insulin resistance in obese patients and the associated disease cluster of type 2 diabetes mellitus (T2DM), hyperlipidemia, and hypertension are major global health problems. Obesity is associated with chronic, low-grade inflammation, known as *metabolic inflammation* or *metaflammation* [1], which is considered a pivotal point in the initiation and progression of insulin resistance and T2DM. Mitochondrial dysfunction induced by oxidative stress contributes to obesity-related insulin resistance [2–4], but the relationship between mitochondrial dysfunction and the pathogenesis of insulin resistance is unknown. Damage to mitochondrial DNA (mtDNA) may disrupt transcription of proteins encoded by mtDNA that are essential for energy metabolism, initiate apoptotic cell death, and alter mitochondrial redox signaling [5–9]. In support of the concept that oxidative mtDNA damage contributes to T2DM, we previously showed that damage to mtDNA increases mitochondrial oxidative stress and insulin resistance in skeletal muscle cell [10,11]. Moreover, in a mouse model of insulin resistance induced by a high-fat diet, we showed that mtDNA damage is associated with mitochondrial dysfunction and increased oxidative stress in skeletal muscle and liver [12]. Fragments of mtDNA known as mtDNA *damage-associated molecular patterns* (DAMPs) may be intercellular mediators of inflammation [13,14]. Such mtDNA fragments are released into the circulation after injury or sepsis and are believed to propagate damage from the initial site of injury or infection to distant organs [15,16]. Inflammation may be propagated by mtDNA DAMPs via activation of one or more pro-inflammatory nucleic acid receptors, including the toll-like receptor 9 (TLR9), NLRP3 inflammasome, and cyclic guanosine monophosphate–adenosine monophosphate synthase–stimulator of interferon genes (cGAS-STING) [13–16].

Since obesity is associated with metainflammation the major goal of the current study was to determine whether obese T2DM patients display elevated contents of plasma mtDNA and whether plasma mtDNA correlates with insulin resistance. Our results comprise the first preliminary evidence in a small group of obese, predominantly women patients, that increased levels of plasma mtDNA fragments correlate with the degree of insulin resistance in obese T2DM patients. Furthermore, obese T2DM patients have significantly increased mtDNA damage and oxidative stress markers in skeletal muscle, which was accompanied with increased systemic inflammation. This study suggests there may be novel therapeutic strategies for reducing insulin resistance and for the design of new biomarkers to measure insulin resistance in humans.

## Methods

### Subjects

We recruited 10 obese (body mass index >35 kg/m^2^) T2DM patients who had hemoglobin A_1C_ levels > 6.5% and a diagnosis of T2DM based on fasting plasma glucose level > 126 mg/dL or current treatment with any oral hypoglycemic drug. De-identified obese diabetic patients were participants in an ongoing research project conducted by WOR in the Department of Surgery, University of South Alabama College of Medicine. We recruited 12 volunteer healthy control (HC) subjects without obesity (body mass index < 30 kg/m^2^) or T2DM from the general community. All subjects were sedentary. All human studies including the source study for recruited T2DM patients were conducted according to the principles of the Declaration of Helsinki and approved by the Institutional Review Board (protocols #10–131, 11–150) of the University of South Alabama. All human subjects gave informed written consent.

### Metabolic parameters and muscle biopsy

Each subject had a medical history, physical examination including measurement of blood pressure and waist circumference, and blood sampling for screening laboratory tests. On the day of the blood sampling and muscle biopsy, subjects reported to the laboratory after an overnight fast (12 h). Peripheral blood (16 mL) was collected into two sterile density gradient tubes (Vacutainer with Ficoll-Hypaque solution, Becton Dickinson, Franklin Lakes, New Jersey). Blood was fractionated by centrifugation at 1,500g for 30 min at 21°C with a swinging bucket rotor. The plasma (upper) fraction was carefully transferred into 15 ml conical tube, frozen immediately, and stored at -80°C until analyzed. Plasma glucose concentration was determined by the glucose oxidase reaction (Glucose Oxidase Analyzer, Beckman, Fullerton, California), and plasma insulin concentration was measured by radioimmunoassay (Coat-A-Count, Diagnostic Products Corp., Los Angeles, California). Homeostasis model assessment for insulin resistance index (HOMA-IR) was calculated using the formula: HOMA-IR = [glucose (nmol/L)X insulin (μU/mL)/22.5], using fasting values. Hemoglobin A_1C_ percentage was measured using a standard kit. Tumor necrosis factor alpha (TNF-α) concentration (Human TNF alpha PicoKine ELISA Kit, RayBiotech, Norcross, Georgia) and lipid profiles were measured using commercially available methods (VAP Cholesterol Test, Atherotech Diagnostics Lab, Birmingham, Alabama).

Samples of skeletal muscle (100–150 mg) were obtained from the vastus lateralis by percutaneous needle biopsy under sterile conditions with local anesthesia (lidocaine, 1%). The muscle samples were immediately dissected free of any adipose and connective tissue under low-magnification microscopy. Muscle samples were snap frozen in liquid nitrogen for DNA and protein analysis.

### Analysis of plasma mtDNA

One T2DM patient and one volunteer from HC were excluded from the analysis of plasma mtDNA since the plasma sample was missing or it was not a sufficient amount of plasma for these patients, accordingly. Thus, we isolated plasma DNA fractions from 9 T2DM patients and 11 volunteers from the HC group (presented in Table 1) and performed quantitative real-time polymerase chain reaction (qRT-PCR) as described previously [17], with minor modifications. Plasma DNA was isolated using a kit (DNeasy, Qiagen, Hilden, Germany) from aliquots of plasma samples (200 μL) that had been frozen at -80°C. DNA was eluted with deionized water (100 μL), and equal amounts of eluant (5 μL) were used for qRT-PCR (VeriQuest Fast SYBR Green qPCR Kit, Affymetrix, Santa Clara, California), according to the manufacturer’s protocol. All primers were designed by Beacon program (Beacon Designer, Premier Biosoft, Palo Alto, California) to amplify 100–200 bp sequences within the specific sequences of the experimental mitochondrial genes. MtDNA sequences quantified included the displacement loop (D-loop) transcriptional regulatory region, which is the transcriptional regulatory of the genome and is particularly prone to oxidative injury [18] as well as regions encoding cytochrome *C* oxidase subunit I (Cox1), and NADH dehydrogenase subunit 4 (ND4). ND4 lie within the mtDNA “common deletion” sequence, a 4,977-bp region of the mitochondrial genome whose deletion is frequently associated with various pathologies [19], whereas Cox1 and D-loop flank either side of the deleted sequence. The abundance of nuclear DNA (nDNA) in each sample was quantified using sequences of the housekeeping gene 18S rRNA. Sequences of primers are provided in Table 1. The qRT-PCR results were normalized by the mean value of 1 HC sample set to 1 and presented as arbitrary units (AU).

**Table 1:** Primers for qPCR detection of plasma mtDNA and nDNA sequences.

Gene	Forward	Reverse
ND4	5’-ACATCCTCATTACTATTCTG-3’	5’-TTAGTGGGAGTAGAGTTT-3’
Cox1	5’-TCATCTGTAGGCTCATTC-3’	5’-GGCATCCATATAGTCACT-3’
D-loop	5’-ATCAACCTTCAACTATCA-3’	5’-ACTGTAATGTGCTATGTA-3’
18S rRNA	5’-TAGAGGGACAAGTGGCGTTC-3’	5’-CGCTGAGCCAGTCAGTGT-3’

### Mitochondrial DNA damage analysis

Damage to mtDNA was assessed as described previously [12], with minor modifications. Skeletal muscle was homogenized in liquid nitrogen, and total DNA was isolated with a kit (DNeasy, Qiagen). DNA was digested with BamHI and quantified. Quantitative alkaline Southern blot was performed to evaluate changes in the density of mtDNA lesions using human mtDNA specific probe (cytochrome c oxidase, subunit I) as previously described [7]. Hybridization images were scanned and band intensities were normalized by the mean value of 1 HC sample set to 1 and presented as AU.

### Protein isolation, Western blot, and oxidative protein carbonylation

For protein isolation from total cellular fractions, frozen skeletal muscle were homogenized in liquid nitrogen and incubated in the lysis buffer (Cell Lysis Buffer, Cell Signaling, Beverly, Massachusetts) supplemented with 0.1 mg PMSF and a 1:100 dilution of protease and phosphatase inhibitor cocktails (Sigma, St. Louis, Missouri). Homogenization was repeated, samples were centrifuged for 10 min at 14 000 *g*, and the supernatants were used for Western blot as described previously [10–12] with the antibodies against PTEN-induced putative kinase 1 (PINK1) (Abcam, Cambridge, Massachusetts) and actin (Sigma, St. Louis, MO). The complexes that formed were detected with horseradish peroxidase conjugated anti-mouse or anti-rabbit IgG antibodies (Promega, Madison, Wisconsin) using chemiluminescent reagents (SuperSignal, Pierce, Rockford, Illinois). An oxidative protein carbonylation assay was performed as described previously [12]. Where indicated, the resultant band images were scanned and analyzed (Fujifilm Image Gauge Version 2.2 software, Fuji, Tokyo, Japan). For protein carbonylation, summarized band intensities for 2 major bands (~50 kDa and ~40 kDa) were normalized by the mean value of 1 HC sample set to 1 and presented as AU. For PINK1 protein content, data were normalized to the density of actin bands and presented as AU.

### Statistical analysis

Data are reported as mean ± standard error of the mean (SEM) and analyzed using statistical software (GraphPad Prism version 7.02 for Windows, GraphPad Software, La Jolla, California). For parametric analysis, differences between groups were assessed using unpaired 2-tailed *t* test. For indicated sets of data, groups were compared using nonparametric analysis (Mann-Whitney test). Correlation analysis (Spearman test) was used to evaluate the relations between mtDNA fragments in plasma and insulin resistance (HOMA-IR). Statistical significance was defined by P <0.05.

## Results

### Metabolic parameters

In contrast to HC subjects, the T2DM patients were markedly insulin resistant (high HOMA-IR index) and had markedly increased fasting plasma glucose, hemoglobin A_1C_, insulin, and triglyceride levels (Table 2).

**Table 2:** Clinical and metabolic data of patients*.

Characteristic	Healthy control	Obese T2DM	*P*
**Age, M/F**	**40.17** **±** **4.47 (6/6)**	**47** **±** **1.9 (2/8)**	**0.16**
**BMI (kg/m**^**2**^**)**	**23.93** **±** **0.73**	**44.71** **±** **1.95**	**<0.0001**
**Plasma glucose (mg/dl)**	**81.58** **±** **3.12**	**139.5** **±** **10.08**	**<0.0001**
**Hemoglobin A**_**1C**_ **(%)**	**5.43** **±** **0.06**	**8.46** **±** **0.48**	**<0.001**
**Plasma Insulin (μU/ml)**	**10.8** **±** **1.73**	**32.39±** **12.00**	**0.06**
**HOMA (IR)**	**2.13** **±** **0.31**	**11.06** **±** **4.26**	**<0.05**
**Plasma Triglycerides** **(mg/dl)**	**84.42±** **12.25**	**172.8** **±** **27.22**	**<0.01**
**HDL cholesterol (mg/dl)**	**64.58** **±** **4.62**	**40.60** **±** **2.5**	**<0.001**
**LDL cholesterol (mg/dl)**	**109.3** **±** **7.66**	**118.0** **±** **14.13**	**0.57**

*Values are given as mean ± SEM.

***Abbreviations***: BMI, body mass index; HDL, high-density lipoprotein; HOMA-IR, homeostasis model assessment for insulin resistance; LDL, low-density lipoprotein.

### Plasma mtDNA fragments and systemic inflammation are increased in obese T2DM patients

**Fig 1:**
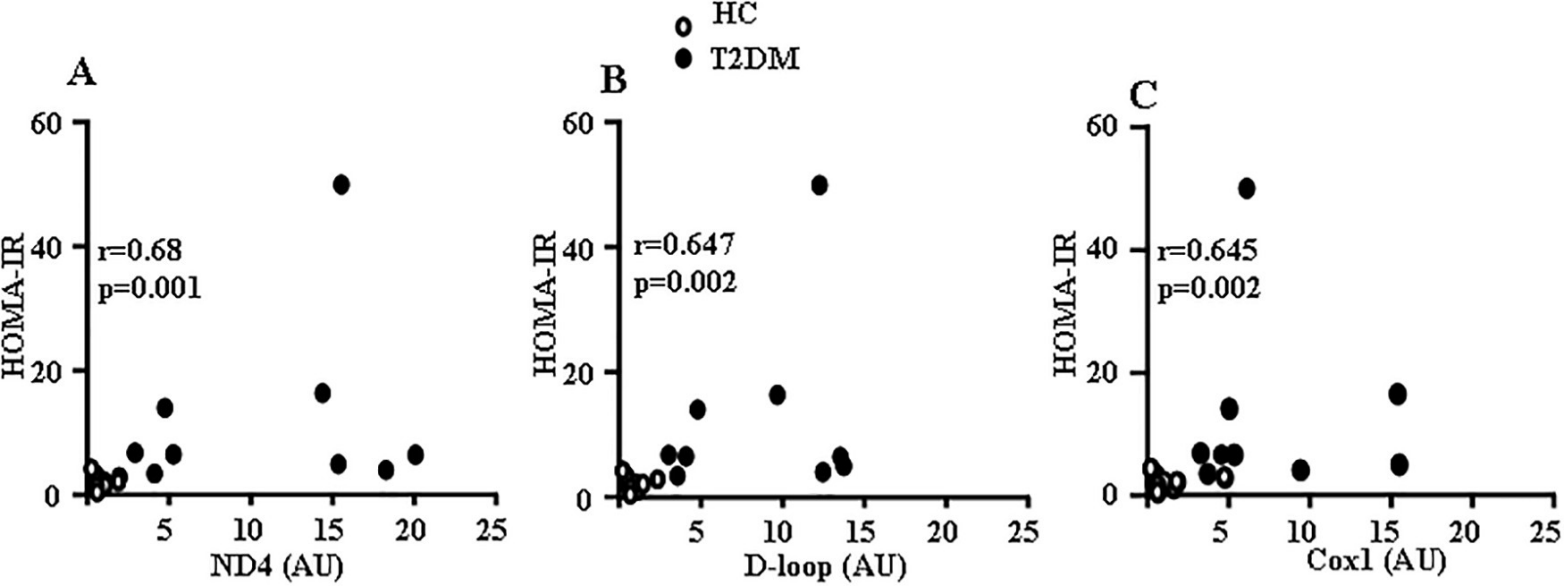
Relationship between plasma mtDNA and HOMA-IR. Significant positive correlation was observed between plasma levels of ND4 (A), D-loop (B) and Cox1 (C) sequences and HOMA-IR (n = 9–11 subjects per group).

**Fig 2:**
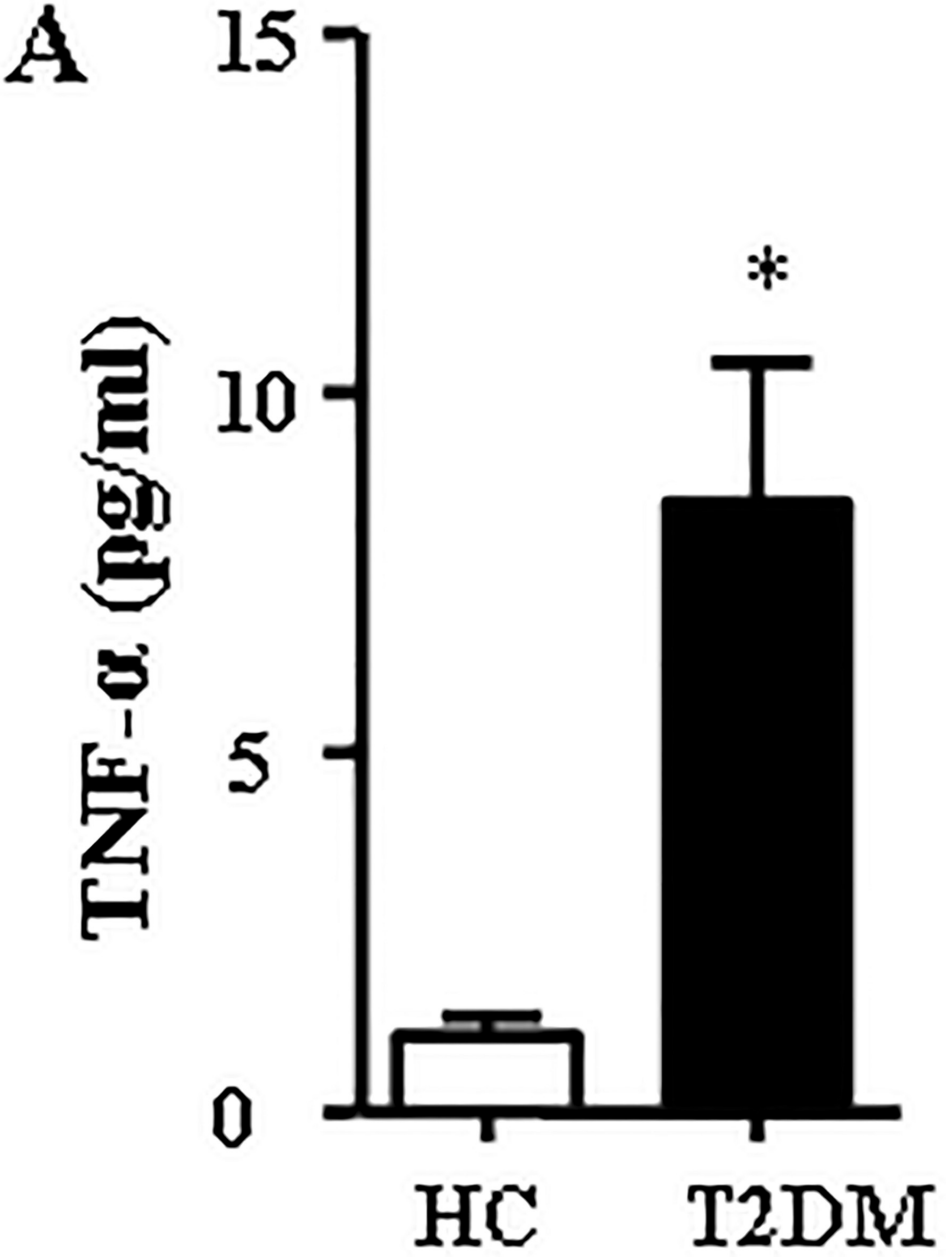
Increased *s*ystemic inflammation in obese T2DM patients. Level of plasma tumor necrosis factor alpha (TNF-α) was significantly greater in obese T2DM patients than HC (mean ± SEM; n = 5–8 subjects per group). *P <0.05, Mann-Whitney U-test.

First, plasma levels of the Cox1, ND4, and D-loop fragments from mtDNA regions were significantly greater in obese T2DM patients compared to HC subjects (Table 3). This suggested selective release of mtDNA and not cell death because nDNA levels in plasma (18S rRNA) were similar between both groups. There was a significant positive correlation between levels of Cox1, ND4, and D-loop plasma mtDNA sequences and insulin resistance quantified by HOMA-IR, despite the high variability in T2DM samples (Fig 1A and 1B). The strongest correlation was observed for the ND4 sequence (Fig 1A). Importantly, plasma TNF-α levels were significantly greater in obese T2DM patients than HC subjects (Fig 2).

**Table 3:** Levels of plasma DNA fragments in patients*.

Gene	Relative fold expression (AU), Healthy control (n = 11)	Relative fold expression (AU), Obese T2DM (n = 9)	*P*
**ND4**	**0.86** **±** **0.17**	**11.18** **±** **2.27**	**< 0.0001**
**Cox1**	**1.156** **±** **0.39**	**7.60** **±** **1.59**	**0.0004**
**D-loop**	**0.89** **±** **0.17**	**8.56±** **1.54**	**< 0.0001**
**18S rRNA**	**1.55** **±** **0.57**	**1.99** **±** **0.93**	**0.67**

*Values are given as mean ± SEM.

### Mitochondrial DNA damage, protein carbonylation, and PINK1 expression

**Fig 3:**
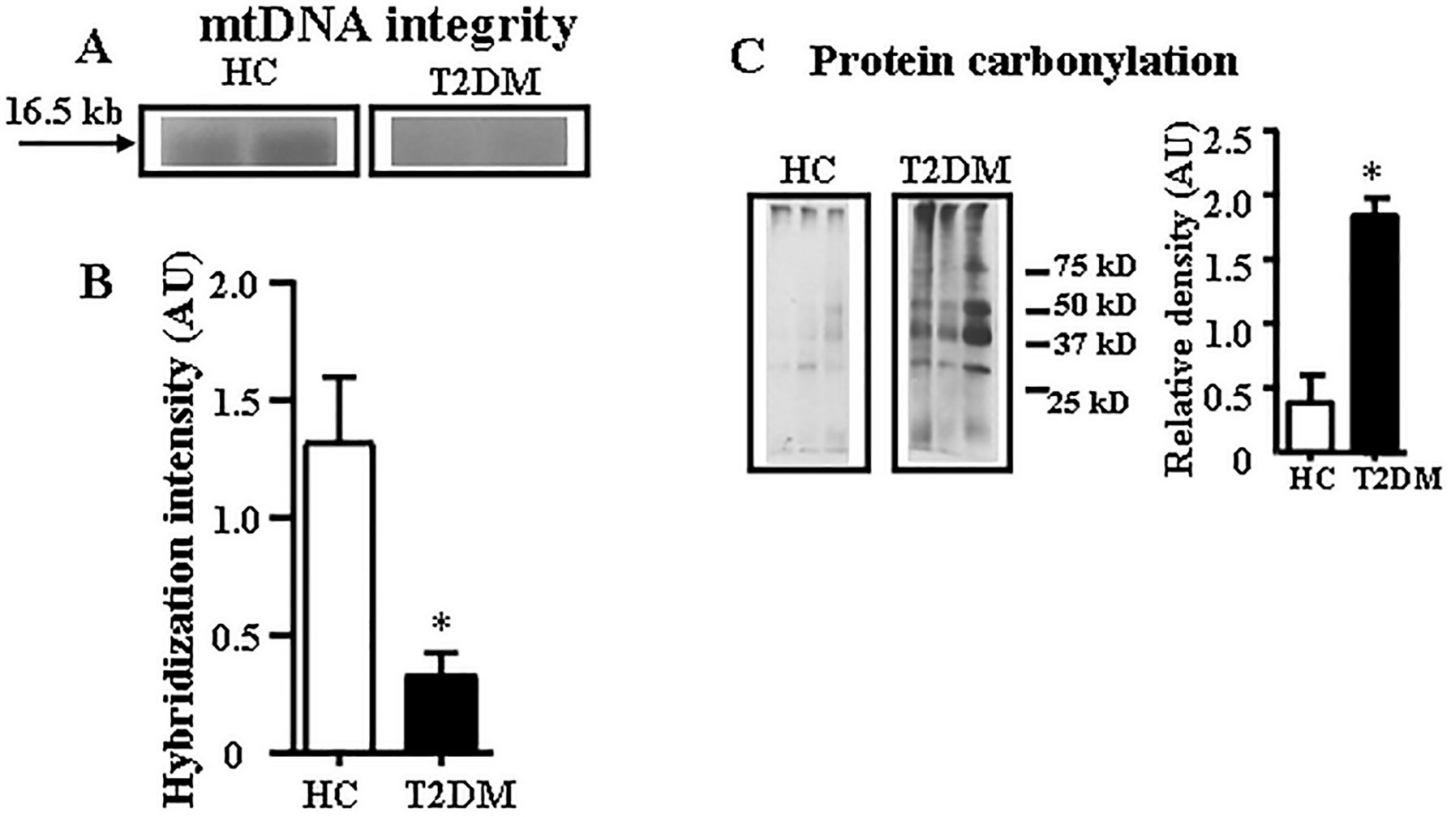
Mitochondrial DNA damage and oxidative protein carbonylation in obese T2DM patients. (A) Representatives of Quantitative Alkaline Southern blot showing that mtDNA damage was greater in obese T2DM patients than HC. In T2DM patients, there was diminished intensity in the 16.5-kb major restricted band (arrow), indicating that DNA breaks caused smaller fragments that migrated further down the gel, with fewer residual full-size restriction fragments. Representatives from different films are presented. (B) Quantitative alkaline Southern blot densitometry showing lower hybridization intensity, indicative of greater mtDNA damage, in T2DM than HC subjects (mean ± SEM; n = 4–5 subjects per group). *P <0.05, Mann-Whitney U-test. (C) Left side-representatives of oxidative protein carbonylation in skeletal muscle from T2DM and HC. Representatives from one film are presented. Right side represents the graph after densitometric quantitation of data. The average results ± SEM are shown. n = 5–6 per group, *P < 0.05, Mann-Whitney U-test.

**Fig 4:**
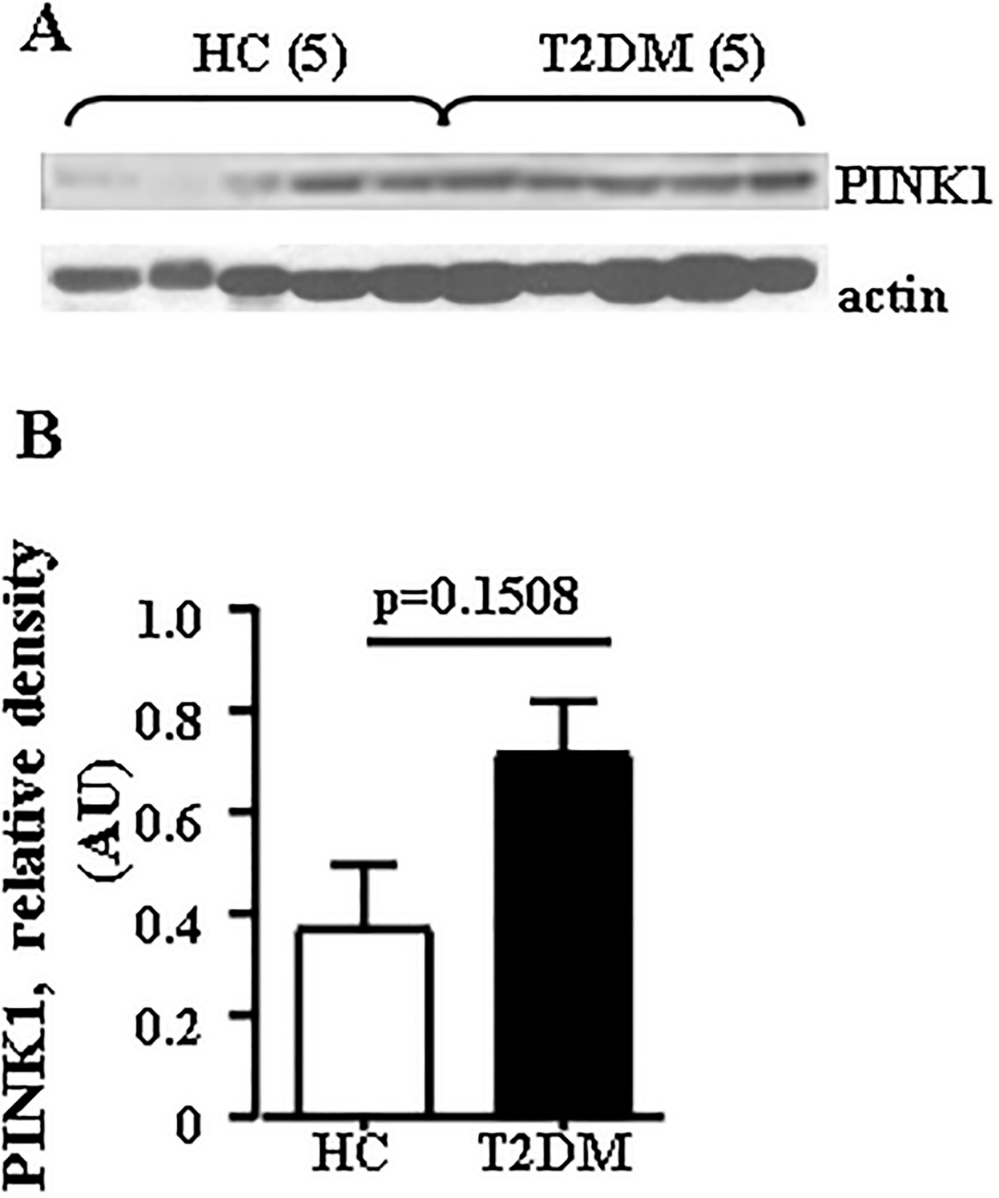
Levels of PINK1 in skeletal muscle in obese T2DM patients. (A) Western blot shows PINK1 and actin bands in 5 T2DM patients and 5 HC subjects. (B) Densitometric quantitation of data normalized to the density of actin bands and presented as AU. The average results ± SEM are shown, (n = 5 per group). P = 0.1508, Mann-Whitney U-test.

Next, we showed that in skeletal muscle, T2DM patients had significantly greater mtDNA damage (Fig 3A and 3B) and protein carbonylation level (Fig 3C) than HC subjects. Since inhibition of mitophagy is a plausible mechanism driving mtDNA fragments elevation in the circulation (*vide infra*), we next compared the content of phosphatase and tensin homologue-induced kinase 1 (PINK1), an established marker of mitophagy [20] in skeletal muscle isolated from both groups. Contrary to our expectation, there is a trend toward increase in abundance of PINKI in skeletal muscle biopsies from obese T2DM subjects (P = 0.1508, Mann-Whitney U-test, Fig 4B), suggesting increased mitophagy in skeletal muscle in T2DM.

## Discussion

This is the first observational report to provide evidence that increased levels of mtDNA fragments in plasma may be associated with insulin resistance in obese human subjects. We showed that plasma levels of mtDNA are elevated in a small group of obese predominantly women T2DM patients, and that there is a significant positive correlation between elevated levels of plasma mtDNA fragments and insulin resistance in humans. Furthermore, mtDNA damage and oxidative stress in skeletal muscle, as well as systemic inflammation were increased in obese T2DM patients.

A recent study showed that obesity-induced DNA released from adipocytes stimulated chronic adipose tissue inflammation and insulin resistance [21]. Although this study did not specify the origin (nDNA or mtDNA) of cell-free DNA. Also, previous studies showed that mtDNA is present in the circulation of healthy human subjects and patients with T2DM, but no quantitative analysis was provided for comparison of plasma levels of mtDNA between T2DM and healthy patients [22]. In the present study, we performed quantitative analysis of plasma mtDNA sequences by using qRT-PCR. The previous study also showed the presence of the mt3243 mutation in the circulatory mtDNA samples only from T2DM patients [22]. Further quantitative studies are justified to evaluate whether there may be a preference for mutated mtDNA sequences entering the circulation.

While a complete understanding of obesity and its metabolic consequences has yet to be achieved, increased oxidative stress in skeletal muscle has recently been proposed as a unifying mechanism promoting mitochondrial dysfunction, lipid accumulation, and insulin resistance [2–4]. Complementing this concept, it has also been suggested that mitochondrial superoxide production is a central component of insulin resistance, including high fat diet-induced insulin resistance in skeletal muscle [4]. Although limited information is available about the molecular triggers for these events, the present results suggest that damage to mtDNA may be an important trigger for insulin resistance, given that mtDNA is more sensitive to damage than nDNA [23]. In addition, we showed that increased mtDNA damage was associated with increased protein carbonylation, a marker for oxidative stress.

Mitochondrial DNA is highly specialized and encodes for proteins essential for energy metabolism. Previously, it had been demonstrated that depletion of mtDNA caused impaired glucose utilization and insulin resistance in skeletal muscle cells, suggesting a crucial role for mtDNA in the development of insulin resistance [24]. In agreement with the notion of a destructive role for reactive oxygen species (ROS) on mtDNA in insulin resistance and T2DM is the finding that there are increased mtDNA mutations and reduced mtDNA content in skeletal muscle from T2DM patients [25, 26], consistent with the hypothesis that ROS may degrade mtDNA in patients who have insulin resistance and T2DM. Additionally, a recent study showed that intracellular oxidative stress increases in skeletal muscle and is associated with an increase in oxidation of mtDNA and a decrease in cytochrome b transcription in a rat model of T2DM [27]. Oxidized mtDNA might not transcribe enough cytochrome b that impairs the function of complex III of the electron transport chain leading to an increase in ROS production in a vicious cycle [27]. Altogether this vicious cycle of ROS generation might be involved in the development of insulin resistance in skeletal muscle and further T2DM [27]. In agreement with this study [27], our results also show that obese T2DM patients have significantly increased mtDNA damage in skeletal muscle, providing observational evidence linking mtDNA damage to insulin resistance.

Numerous studies reported an increase in circulating mtDNA fragments in various human diseases, aging and in conditions with acute tissue injury, such as trauma, myocardial infarction, sepsis, and intensive care unit [13, 14,17, 28–33]. Interestingly, recent work showed an increase in circulating mtDNA fragments in coronary heart disease patients without T2DM compared to healthy patients and a consistent increase of circulating mtDNA fragments in coronary heart disease patients with T2DM compared with those without the diseases [34]. The present results extend these findings, since we showed elevated levels of mtDNA sequences in the circulation of obese T2DM patients compared to healthy nondiabetic subjects without obesity.

The origin of plasma mtDNA and mechanisms involved in the increase in plasma mtDNA abundance are unknown. Possible mechanisms of accumulation of mtDNA in plasma may include the release of mtDNA from cells of varied origin because of depletion of mitochondrial fission or mitochondrial permeability pores [13], cell death in necrosis or apoptosis, or mtDNA release without degradation during autophagy [29]. In addition to passive release of mtDNA from the cells, some cells deliver mtDNA into circulation actively. Thus, mtDNA in plasma may be associated with a different kinds of subcellular or molecular particles, including mitochondria derived vesicles, exosomes and microparticles [35]. Boudreau et al. showed that activated platelets secrete functional intact mitochondria into the extracellular space as free organelles or incorporated into microparticles [36]. They showed that released mitochondria could be hydrolyzed by phospholipase A2-IIA-releasing mtDNA, thus promoting leukocytes activation [36]. Also, neutrophils are spontaneously releasing mtDNA in the circulation as a part of neutrophil extracellular traps in absence of cell death or membrane disruption [37]. Moreover, a recent study demonstrated that red blood cells homeostatically bind and scavenge mtDNA and thus prevent lung injury [38]. Additional studies are needed to further characterize the origins and size of disease-related forms of circulating mtDNA fragments in plasma of obese T2DM patients.

Regarding our study, most likely inhibition of autophagy was not involved in the release of mtDNA, because the level of PINK1 tended to increase in T2DM patients, rather indicating increased mitophagy in skeletal muscle of T2DM patients. Since mtDNA is more sensitive to oxidative stress than nDNA [23], it is possible that formation of plasma mtDNA fragments in obese T2DM patients may be triggered by molecular fragmentation induced by oxidative stress, followed by their release into the circulation [39]. In this regard it is interesting, that the plasma mtDNA fragments within the ND4 sequence have the strongest correlation with insulin resistance; in addition, ND4-mtDNA showed the highest level in plasma from obese T2DM patients. The ND4 gene is located within the mitochondrial “common deletion” which is prone to oxidation and is associated with a variety of diseases [19]. The second strongest correlation was shown for D-Loop transcriptional regulatory region, which is also specifically prone to oxidation [18]. The lowest correlation was observed for Cox1 sequence which flanks the “common deletion” area. Although requiring further investigation, these observations support the idea that abundance of plasma mtDNA in obese T2DM group may represent the increased degradation and release of selective sequences under oxidative stress. Degradation of mtDNA occurs under conditions of increased oxidative stress [40], but the fate of the degraded DNA is poorly understood. In addition, endogenously oxidized mtDNA causes inflammatory responses *in vitro* and *in vivo* [41,42]. Mitochondrial ROS and oxidized mtDNA may activate the NLRR3 inflammasome and TLR9 signaling [41–43]. Based on the recent findings that NLRP3-associated inflammasome is upregulated in T2DM patients [44] and instigates obesity-induced inflammation in insulin resistance [45, 46], we have started to evaluate whether plasma mtDNA is oxidatively damaged in T2DM patients. Future studies will be required to define the cascade of events that increase plasma mtDNA and the signaling pathways that are involved in the obesity-related inflammatory responses to plasma mtDNA.

Limitations of the present study include the sex difference between the groups of T2DM and HC subjects (Table 1), and we cannot exclude the effect of sex on the observed results. In addition, a limited number of subjects were included, and we did not evaluate non obese T2DM patients and nondiabetic obese patients. Future studies with larger groups of age-, sex-, and comorbidity-matched individuals may resolve these uncertainties in the present study.

The results of the present study justify the evaluation of plasma mtDNA as a potential cost-effective biomarker for detection of insulin resistance in patients. Insulin resistance may be subtle in the early stages, and diagnosis may be delayed until symptoms are evident and end organ damage has occurred. Current methods to test insulin sensitivity in humans include the euglycemic hyperinsulinemic glucose clamp, and measurement of steady-state plasma glucose are direct, but time and resource-intensive approaches. Alternative surrogate approaches, including calculations based on fasting plasma insulin and glucose levels, or analyses of glucose and insulin levels after intravenous or oral glucose administration; are useful but provide only modest correlation with the primary analyses [47, 48]. It is important to note, that determination of mtDNA abundance in plasma is less costly, invasive and laborious than above mentioned direct assessments of insulin resistance. Further studies may determine whether the levels of circulating, cell-free mtDNA might be a proxy for insulin resistance severity and a cost-effective, early, and reliable marker for the prognosis and diagnosis of insulin resistance, and whether pharmacologic strategies to lower the circulatory levels of mtDNA may prevent insulin resistance and metabolic syndrome.

## Supporting information

S1 Dataset Excel sheet of dataset on which the conclusions of this manuscript were made. (XLS)
